# Pulmonary Amyloidoma: A Case Report and Brief Review of the Literature

**DOI:** 10.3390/diagnostics13223411

**Published:** 2023-11-09

**Authors:** Matache Radu Șerban, Savu Cornel Florentin, Constantin Ancuta Alina

**Affiliations:** 1 Department of Cardio-Thoracic Pathology, “Carol Davila” University of Medicine and Pharmacy, 050474 Bucharest, Romania; 2 Institute of Pneumology “Marius Nasta”, 050159 Bucharest, Romania

**Keywords:** nodules, lung biopsy, amyloidoma, Congo red staining

## Abstract

We report the case of a 59-year-old female patient, a former smoker, who was diagnosed with bilateral pulmonary nodules. Extensive medical investigations were conducted, including a surgical lung biopsy, which led to the diagnosis of pulmonary amyloidoma. The diagnostic process was guided by the presence of a persistent, polymorphic, and nonspecific clinical picture, strengthened by imaging findings characterized by mixed nodular lesions and the addition of interstitial involvement, along with partial deterioration of the pulmonary parenchyma architecture. Although it is recognized as a benign tumor, pulmonary amyloidoma requires special care in order to rule out systemic involvement, association with lymphomas, or systemic amyloidosis. This case highlights the comprehensive investigations required in the presence of multiple pulmonary nodules and the wide range of possible diagnoses. It underscores the pivotal role of surgical lung biopsy and histopathological examination. The case is instructive, addressing a rare pathology, on the border between specialties, while also emphasizing potential evolving challenges and providing further insights into the clinical course of this disease.

**Figure 1:**
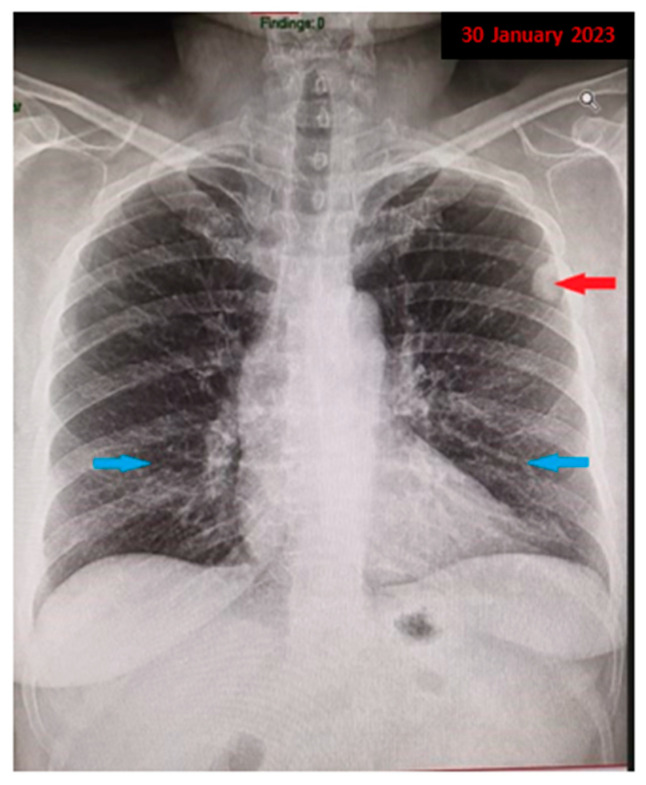
We present the case of a 59-year-old female patient, a former smoker with a history of 15 pack-years, who quit smoking 16 years ago, with prolonged occupational exposure to respiratory irritants (hairdresser for 23 years), who presented to our medical department for a detailed investigation of a lung nodule identified following examinations conducted at a regional medical facility. Her medical history up to this point showed no significant issues, except for a previous diagnosis of renal cysts and chronic treatment with antihypertensive medications (diuretics, angiotensin-converting enzyme inhibitors—ACE inhibitors, and central antihypertensives) for hypertension. In December 2022, she went to a regional medical care unit reporting mixed-type cough, predominantly irritative, with paroxysmal nocturnal exacerbations, moderate-effort dyspnea, and fatigue. Initially, symptomatology was labeled as residual from an acute upper respiratory tract infection. A chest X-ray was performed (Figure 1), which revealed a nodular pulmonary opacity (red arrow) of approximately 20/15 mm, located peripherally in the left upper lobe (LUL), adherent to the pleura, and an accentuated pulmonary interstitial pattern basal, bilaterally (blue arrow). Further, she was recommended to perform a spirometry with a bronchodilator test and additional imaging investigations with a contrast-enhanced chest computed tomography (CT) for accurate diagnostic assessment of the radiologically detected pulmonary nodule.

**Figure 2:**
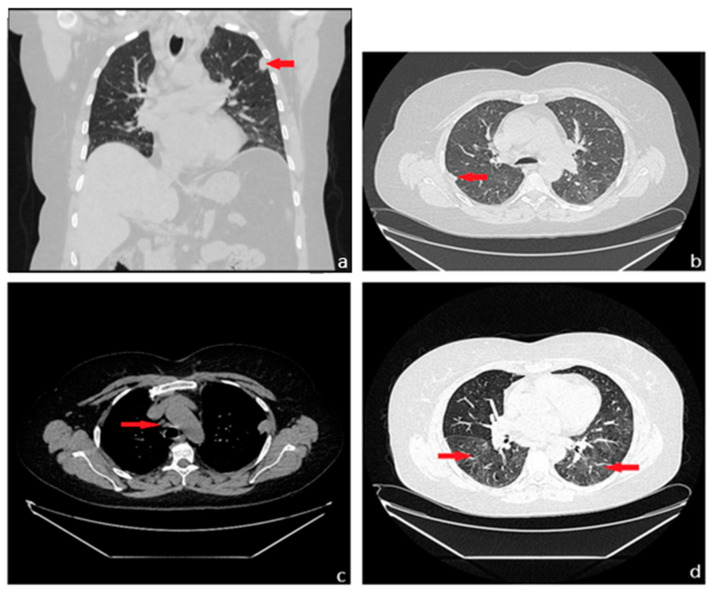
A chest CT was performed, which revealed subpleural micronodular and nodular pulmonary lesions located in the upper lobes bilaterally, up to 13/22 mm ((**a**): red arrow), associating thickening of the interstitial septa, acquiring a right subpleural pseudo-nodular appearance ((**b**): red arrow). Additionally, mediastinal lymphadenopathy was observed in Barety’s space, measuring approximately 10 mm ((**c**): red arrow), and pulmonary interstitial involvement with a ground-glass appearance in the lower lobes ((**d**): red arrow). The nonspecific clinical context, associated with the presence of observed imaging changes, prompted further investigations, raising suspicion of diffuse interstitial lung disease. Meanwhile, the patient’s pulmonary function tests indicated normal values, but with a slight decrease in the diffusing capacity of the lung for carbon monoxide (DLCO), with an estimated DLCO value of 72% of the predicted values. Additionally, the patient underwent a fiberoptic bronchoscopy examination, revealing bilateral bronchial secretions, otherwise normal laryngeal dynamics, and no apparent proliferative elements or active lesions of the mucosal tissue in the examined areas. The microlavage of the left upper lobe bronchus was evaluated for bacteriological exams and did not yield any pathogenic micro-organisms. Ziehl–Neelsen staining from the lavage liquid also revealed no acid-fast bacilli. Despite the extensive and thorough investigations conducted previously, the etiology of the polymorphic pulmonary lesions could not be established.

**Figure 3:**
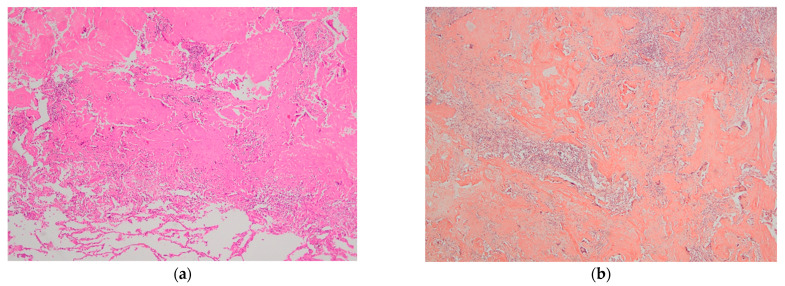
In March 2023, the patient was referred to the Thoracic Surgery Clinic for a pulmonary biopsy to achieve a definitive diagnosis and subsequently determine the appropriate therapeutic approach. Under general anesthesia and selective orotracheal intubation, a left video-assisted thoracoscopy (VATS) was performed, and a wedge resection of two pulmonary nodules located in the left upper lobe was carried out. The patient’s postoperative course was favorable, marked by a gradual recovery, with stable hemodynamic and respiratory parameters maintained through the administration of antibiotics, anti-inflammatory drugs, and prophylactic anticoagulants. Since minimal pleural drainage persisted with serosanguineous fluid, the chest tube was removed on the second day postoperatively, under clinical and radiologic control, ensuring adequate aeration and documented hemostasis. The histopathological examination revealed fragments of pleuro-pulmonary tissue measuring 3 cm in length, exhibiting a yellowish color with an area of light brown condensation, measuring 10 mm along its long axis. Fragments of lung parenchyma with nodular formation were described, represented by deposits of amphophilic eosinophilic material, delimited by multinucleated giant cell reaction and chronic inflammatory infiltrates. Also, the laboratory described lung tissue with architecture modified by nodular deposits of amyloid with glassy eosinophilic appearance and foreign-body giant cell histiocytes evident in the bottom of the image; HE staining, 40× (**a**). Congo red staining, 40× (**b**) revealed the salmon pink color of the amyloid under light microscopy, supporting the diagnosis of pulmonary amyloidoma. Corroborating the clinical–paraclinical aspects, from this point, it was considered that interdisciplinary collaboration was mandatory; so, a series of complementary medical consultations, with a diagnostic and therapeutic purpose, was recommended.

After assessment by hematology and rheumatology evaluations, further investigations were recommended in order to rule out systemic amyloidosis or other underlying causes.

− The white blood count (6.0 × 10^3^/uL) and the red blood count (4.5 × 10^6^/uL) had normal values.
− Serum protein electrophoresis with immunofixation did not reveal monoclonal bands (IgG, IgA, IgM, k or λ chains).
− Urinary protein electrophoresis identified minimal proteinuria (0.06 g/24 h) and the absence of kappa and lambda chains.
− The C-reactive protein (CRP) value (5.2 mg/L) was slightly elevated compared to the normal value (5 mg/L), denoting inflammation.
− The serum amyloid had values exceeding twice the upper limit of normal (23 mg/L, normal range < 10 mg/L).
− Serological testing investigations revealed no abnormalities; tests for connective tissue diseases CTD (RF, ANA, aCCP anti-c-ANCA antibodies, anti-P-ANCA antibodies, and serum angiotensin-converting enzyme levels) were negative.
− The abdominal ultrasound reported a well-defined liver (right lobe of 138 mm, left lobe of 59 mm), normal dimensions, clear outline, homogeneous echostructure, and without dilatation of intrahepatic bile ducts. The spleen measured 108 mm, with a homogenous echostructure, regular borderline, no space-occupying formations, no collateral circulation, and a normal-sized splenic vein in the hilum. There were bilateral renal cysts and the left kidney had an irregular outline, ectasia of the pyelocaliceal system, and an appearance of grade 2 ureterohydronephrosis, with no retroperitoneal adenopathies.
− The cardiological consultation revealed quasi-normal findings on the transthoracic echocardiography, with changes secondary to hypertensive cardiomyopathy but with the recommendation to undergo a cardiac MRI to rule out the early onset of cardiac amyloidosis. Unfortunately, this aspect was neglected and delayed due to the complications that occurred in the context of ureterohydronephrosis.
− Troponin T was 8 pg/L (nL < 14), and Natriuretic Peptide Test (NTproBNP) was 45 pg/mL (normal level < 287).

**Figure 4:**
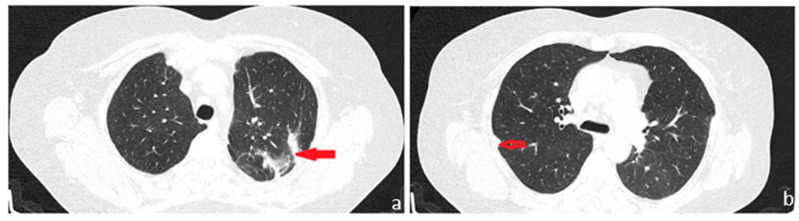
During the patient’s follow-up six months later, she was found to be in good general condition, with minimal respiratory symptoms and a chest CT imaging assessment with no particularities. We believe that the interstitial changes detected in the initial chest CT examination most likely represent the residual signs of an infection that was suppressed while undergoing broad-spectrum antibiotic treatment and symptomatic care, as initially recommended during the initial medical evaluation in the field. Right apical sequelae fibrotic changes ((**a**): red arrow) were observed, consistent with the findings before the surgical intervention, as well as residual postoperative changes ((**b**): red transparent arrow) located in the left subpleural area. Moreover, there was a regression of the ground glass imaging appearance, which was present bilateral, in bases, in March 2023. Thus, the lack of respiratory symptoms and the absence of progression in the imaging lesions represented the arguments for the localized substrate of the condition. Subsequently, immunohistochemistry tests were performed, which confirmed the diagnosis. Although the histopathological diagnosis of pulmonary amyloidoma was established, a comprehensive spectrum of investigations was conducted to exclude systemic damage. The presence of the nonspecific inflammatory syndrome with a high level of CRP and the increased serum titer of amyloid A dictated investigations in search of a secondary amyloidotic substrate. Complementary to the histopathological examination, the performance of immunohistochemistry (IHC) tests, bone marrow biopsy, abdominal fat pad biopsy, and salivary gland biopsies would have been useful during the diagnosis process in order to evaluate systemic lymphoproliferative disorders and plasma cell dyscrasias, but the decision to not undergo all these procedures and the timing of their performance was the patient’s decision. At this moment, the absence of disease progression most likely denotes the indolent character of the disease but does not ensure its benignity and possible distant involvement. The approach chosen is to closely monitor the case, with the patient undergoing periodic interdisciplinary consultations. The current case outlines the diagnostic process for the pulmonary nodular amyloidoma, a rare disorder with only 12 cases reported in the literature [1], established secondary to an exhaustive panel of investigations, triggered by the radiological discovery of a pulmonary nodule. The initial imaging appearance with the nodular mass in the left upper lobe, associated with other discrete interstitial changes in the lower lung areas, bilaterally, imposed a laborious differential diagnosis. Additional hypotheses included fungal infections, benign nodules of various etiologies, neoplastic substrate, or, given that our country remains endemic for tuberculosis, a bacillary etiology. Similar to other reported cases, excisional surgical biopsy was mandatory, as the ‘gold standard’ for diagnosis continues to be the histopathological examination, which demonstrates apple-green birefringence with polarized microscopy after staining with Congo red [2]. The particularity of the case lies in the earliness of the diagnosis, given that the disease was detected in this limited form, with the absence of signs of systemic involvement, although it is not excluded that it may evolve into multi-organ impairment in the future.

Amyloidosis can be extremely heterogeneous and describes a group of diseases characterized by the extracellular deposition of insoluble fibrillar proteins that can originate from serum proteins or be produced locally [2]. An important feature of amyloid proteins is their capacity to infiltrate almost all organs and systems within the body. Since these proteins are deposited extracellularly in the form of amyloid fibrils and cannot be metabolized, they can induce both systemic and localized toxicity [3]. This leads to cell death, subsequent functional impairments, and gradual disruption of the structure, integrity, and function of the tissues [2,4].

The scientific literature indicates that amyloidosis can manifest as a diffuse process with systemic involvement or be localized to a particular organ. The primary organs involved are the kidneys, heart, digestive tract, liver, and skin [5,6]. Respiratory involvement occurs in 50% of the patients with amyloidosis [7] in different clinical and radiological patterns [6]. Depending on the anatomic site of involvement [8], three histopathological patterns are recognized: diffuse alveolar–septal, nodular, and tracheobronchial [4,6,7]. Other classifications have been proposed based on radiographic or bronchoscopic findings [2].

The literature indicates the existence of two amyloid forms with a high potential to affect the respiratory tract. The most common pattern is AL light chain (AL) amyloidosis, with an occurrence consecutive to the deposition of excess immunoglobulin light chain fragments, subsidiarily with a substrate of plasma cell dyscrasia [9,10]. AL amyloidosis accounts for approximately 70% of all systemic amyloidosis cases [9], or 63–80% in other sources [6]. The second type of amyloid is reactive systemic AA amyloidosis, caused by the accumulation of the acute-phase reactant serum amyloid A, which occurs secondary to various chronic inflammatory conditions [11].

The clinical symptoms of respiratory tract amyloidosis are nonspecific and can vary depending on localization. In cases of localized amyloidosis, respiratory symptoms can range from asymptomatic pulmonary nodules to diffuse parenchymal deposits. As in our case, the clinical presentation is often characterized by cough, signs of bronchial obstruction, recurrent lower respiratory tract infections, associating symptoms such as dyspnea or wheezing, which can mimic other pulmonary diseases [2,7,9].

High-resolution computed tomography (HRCT) evaluation is a valuable diagnostic tool, as it can identify features that may resemble various interstitial lung diseases [2,7,12,13]. Therefore, among the vast panoply of CT imaging changes occurring in the lung in the context of amyloidosis, a particular classification draws our attention. Some key signs are described, represented by nodular interlobular septal thickening, smooth interlobular septal thickening, thickening of the bronchovascular bundles, and nodules, most of them calcified. Additionally, there are ancillary signs that encompass features like ground-glass opacity, patchy bilateral consolidations (which can sometimes show calcifications), and, rarely, lung cysts in lymphoproliferative disorders. A third category of changes includes nonparenchymal signs, represented by pleural thickening (associated or not with pleural effusion), hilar and mediastinal lymphadenomegaly with calcification (common in the AL form of amyloidosis but uncommon in the AA variant), and myocardial infiltration (MRI demonstrated) [12,13].

In practice, the focus remains primarily on the histopathological patterns mentioned earlier.

− The diffuse parenchymal or alveolar septal type is characterized by the presence of amyloid deposits in the alveolar septa and vessel walls and represents a rare manifestation of amyloidosis in the lungs, most frequently being associated with systemic AL amyloidosis. The dominant symptom in this form is progressive exertional dyspnea, and the clinical course is typically more severe. HRCT findings result in reticular opacities, interlobular septal thickening, micronodules, and, less frequently, ground-glass opacification, traction bronchiectasis, honeycombing, and sometimes mediastinal lymphadenopathy [2,4,7,14].
− Nodular pulmonary amyloidosis is a consequence of localized AL deposits and is usually an incidental finding on chest radiography. The pattern includes either solitary or, most frequently, multiple well-defined nodules with diameters ranging from 1 cm to 15 cm. These nodules are often peripheral and subpleural, and they tend to occur preferentially in the lower lobes, bilaterally. Slow growth is typically observed, and calcification or cavitation may occur, although these are rare events. The average age of onset is 67 years, with a higher incidence in men [2,4,5,7].
− It appears in the form of multifocal submucosal plaques, with amyloid deposits occurring circumferentially in the submucosal tissue plane of the large airway, sometimes accompanied by calcifications. These deposits can lead to airway narrowing, resulting in symptoms like wheezing, distal atelectasis, recurrent pneumonia, or lobar collapse. In some instances, solitary nodules may be mistaken for endobronchial neoplasia. The peak age of onset is between 50 and 60 years, and it occurs equally in men and women [2,5,7,15].

Another pathological entity is pulmonary amyloidoma, a benign rare tumor, a mass of amyloid protein without evidence of systemic amyloidosis. It is typically discovered incidentally on chest radiographs in asymptomatic elderly patients. Amyloid nodules may be solitary or, more commonly, multiple apparently isolated masses of amyloid deposition and are reported to occur only in AL amyloidosis. However, it always raises the issue of a rigorous differential diagnosis through potential confusion with primary bronchogenic carcinoma or metastatic diseases. Furthermore, excluding systemic involvement is crucial in the management of these patients [1,3,16]. Thoracic amyloidoma can grow to a considerable size while remaining asymptomatic, but ultimately, it can lead to significant neurological and respiratory effects as it infiltrates the spinal cord and lungs. Isolated amyloidoma could grow and invade adjacent tissue through local extension with necrosis. The demonstration of apple-green birefringence with Congo red stain under polarized light is the gold standard for diagnosis [1,2,3], as was performed in our case.

This pathology, at the border between specialties, represents the perfect mime, predisposing to a complex staged differential diagnosis. Given that the case history of the subject is precarious, there is no well-defined guidance in approaching it. The key solution involves multidisciplinary cooperation with a primary focus on ruling out malignant etiology and granulomatous diseases.

## Data Availability

No new data were created or analyzed in this study. Data sharing is not applicable to this article.
